# Surface effects on the self equilibrium, self bending and symmetry lowering of nanofilms

**DOI:** 10.1038/s41598-019-53555-x

**Published:** 2019-11-18

**Authors:** Jiangang Li, Meiqin Han, Lingfang Li, Zhixiang Gao, Huili Zhang

**Affiliations:** 1 School of Physics and Electronic Science, Shanxi Datong University & Shanxi Province Key Laboratory of Microstructure Electromagnetic Functional Materials, Shanxi Datong University, Datong, 037009 P.R. China; 2 Department of Mathematics and Physics, Chengde Petroleum College, Chengde, 067000 P.R. China

**Keywords:** Nanoscale materials, Theory and computation, Nanoscale devices, Nanoscale materials

## Abstract

A continuum theoretical scheme for self equilibrium, self bending and symmetry lowering of nanofilms was obtained by considering surface elasticity, surface stress and the corresponding surface slice thickness. When surface stress and surface elasticity are both balance, the nanofilm is simply compressed (or expanded). When the surface stress or surface elasticity is imbalance, the nanofilm will bend. On the other hand, surface stress and surface elasticity imbalances induce a nanofilm to curl into a nanotube when the nanofilm is very thin. The surface stress and surface elasticity balances induce uniform in-plane strain (the overall film relaxation), while the vertical direction of the nanofilm relaxes reversely due to Poisson’s effect. And then, the crystal lattice constants of in-plane and vertical directions are different from each other, the ratio of these two lattice constants decrease with film thickness increase. Hence, the symmetry of the nanofilm is lowered by the overall film relaxation.

## Introduction

Over the past decades, Nanostructures have been widely used for nanoelectromechanical systems (NEMS)^1–6^. The small scale of nanostructures improves their sensitivity greatly. They can be used as subfemtometer displacement sensing^6,7^, zeptonewton-scale force sensing^8^, zeptogram-scale mass sensing^9,10^, and nanoscale energy harvester or nanogenerator and so on^11–14^. The high sensitivity of nanostructure is charming, but reducing of the scale means that surface effects are more important^15,16^. The surface stress and surface elasticity effects strongly influence their mechanical properties and cause the mechanics of nanofilms to be very different from their bulk material counterpart^17^. In other words, the mechanical properties of nanostructures are strongly size dependent and surface modulated^18,19^. There are many works including continuum theory^20^, atomistic calculation^21^, and experimental research have investigated this phenomenon extensively^22^. This size dependent and surface modulated nature induces nanostructure mechanical properties to be more complex comparing with bulk materials^23,24^.

Surface stress and surface elasticity play an important role in the thermodynamics of solid material surface. They allow us to describe macroscopic phenomena without the knowledge about atomistic progress details. The surface-to-bulk ratio is larger enough to allow the surface stress and surface elasticity to influence the overall mechanical properties of nanostructures. The surface stress is usually expressed as *σ*_s_ = *σ*_s0_ + *c*_s_*ε*_s_^25^. Where *c*_s_ is surface elastic parameter, *σ*_s0_ is intrinsic surface stress and *c*_s_*ε*_s_ serves as surface stress induced by surface strain. Surface stress induces nanofilms to behave very different from the bulk counterpart. Usually, nanofilm surface is not smooth. The surface roughness, surface reconstruction and surface relaxation induce surface area to be very different from the inner core. There are some specific differences between surface area and inner core, including mechanics, surface stress and surface elasticity effects^16^. The surface stress effect induces a nanofilm to behave no longer as a plane. Nanofilms always folds, wrinkles, bends and curls^26^. Without external load ‘surface stress’, solid films will not bend (and will not fold, wrinkle or curl at the same time). In other words, if the surface atomic structure is neglected, the intrinsic surface stress will absence^27^. A smooth surface is obtained, and the nanofilm will exceed a two-dimensional plane (neglect the film thickness here). The existence of non-ignorable atomic structure at nanofilm surface induces intrinsic surface stress and causes nanofilms to bend further more. This surface effect induces the mechanical properties of nanofilms to be very different from micron scale thick films^28,29^. Surface atomic structure undergoes significant reconstruction, and this surface reconstruction induces surface relaxation, the relaxation extends several atomic layers below film surface. On the other hand, the elastic constants of crystals are very sensitive to inter-atomic distance *d*. Researchers pointed out that the elastic constants follow approximately a variation of *d*^-4^ in bulk material^30,31^. The surface stress will not only exist at outermost atomic layer, but also exist at near surface atomic layer and inward the interior of nanofilm. The area where exists surface stress can be called as stress surface slice. Since surface reconstruction changes inter-atomic distance, the elastic properties near surface layers should be changed and different from bulk material counterparts^16^. A nanofilm with reconstructed surface layers can be treated as a composite film with a sandwich structure which is composed of a bulk like core being not influenced by surface effect, and two surface slices being obviously influenced by surface effect. The area whose elastic property is obviously influenced by surface effect can be called as elasticity surface slice. Within elasticity surface slices, the additional Young’s modulus (biaxial modulus) should be introduced in that surface effect changes the elastic constant. Actually, surface stress effect and surface elasticity effect should extend deep into the inner core and it is a gradual progress and fades off slowly^16^. Lin *et al*. studied the Mechanical peeling of van der Waals heterostructures^32^. They discovered a new characteristic length, the elasto-peeling length, that is a crucial parameter that reflects the bending and interfacial properties of the layered materials^32^. The elastic constants of different atomic layers should be different from each other. For the sake of simplicity, the surface elasticity and surface stress within surface slice can be averaged.

With the loading of intrinsic surface stress, nanofilms should bend or expand (or shrink). The equilibrium strain and bending curvature showed size dependent. The surface stress acts on a nanofilm surface, the effect is similar to a pre-tensional adhesive tape on top and bottom surfaces of the film. This surface stress effect induces the lattice constant along in-plane direction to differ from the bulk material counterpart. While, the vertical direction lattice constant is not only different from bulk material but also different from the in-plane direction counterpart. The lattice constant difference between these two directions induces a lowered symmetry of the nanofilm^33,34^. And the intrinsic surface stress makes the bending behavior of a nanofilm be very different from macroscopic thick film counterpart. It changes not only the bending curvature degree but also the bending direction^27^.

In this paper, a continuum theory for self equilibrium, self bending and symmetry lowering of nanofilms was established by considering surface elasticity, surface stress and surface slice thickness. In section 2 the model for the equilibrium state and bending state of nanofilms were established by using the principle of minimum energy. In this section, bending curvature and elongation strain of nanofilms were studied. The application of current theory in some nanofilms is addressed in section 3. The equilibrium strain, bending curvature, and symmetry lowering of nanofilms are discussed in this section. Section 4 summarizes our conclusions finally.

## Theory and Models

For the sake of simplify, we assume that the thicknesses of top and bottom surface slices are equal to each other (both stress and elasticity surface slices). *t*_ts_ = *t*_bs_ = *t*_s_, and *t*_tsσ_ = *t*_bsσ_ = *t*_sσ_, where *t*_ts_ and *t*_bs_ serve as top and bottom elasticity surface slice thicknesses, *t*_tsσ_ and *t*_bsσ_ serve as top and bottom stress surface slice thicknesses, respectively.

**Figure 1:**
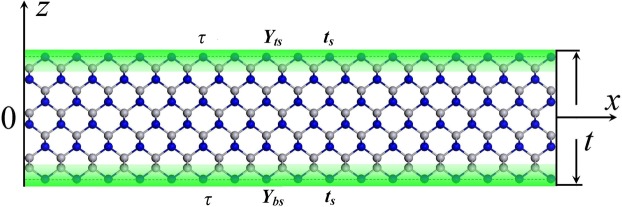
Schematic of a nanofilm with surface slice thickness *t*_s_ and surface Young’s modulus *Y*_s_ (surface biaxial modulus). The blue backdrop part near surface is surface slice with thickness *t*_s_. The *z* axis is fixed along vertical direction and the reference plane is set on the mid-plane of the nanofilm.

The coordinate system was established as shown in Fig. 1. Reference plane is set on mid-plane of the nanofilm and *z* axis is perpendicular to film plane. Top and bottom elasticity surface slice can be expressed as

1 $$\frac{t}{2}-{t}_{s}\sim \frac{t}{2}$$

and

2 $$-\frac{t}{2}+{t}_{s}\sim -\,\frac{t}{2}.$$

And surface Young’s modulus (or biaxial modulus) within elasticity surface slice is

3 $${Y}_{s}=\frac{1}{{t}_{s}}{\int }_{{t}_{s}}{Y}_{s}(z)dz=\frac{1}{{t}_{s}}S.$$

Where surface elasticity

4 $$S={\int }_{{t}_{s}}{Y}_{s}(z)dz={t}_{s}{Y}_{s}.$$

Where *Y*_s_(*z*) is served as surface Young’s modulus (surface biaxial modulus) which is varied with *z* coordinate. *Y*_s_ is averaged surface Young’s modulus (surface biaxial modulus) within elasticity surface slice. It is worth mentioning that surface Young’s modulus (surface biaxial modulus) is additional modulus but not the real modulus within elasticity surface slice. The real Young’s modulus (biaxial modulus) within elasticity surface slice should be constructed by bulk Young’s modulus (biaxial modulus) plus surface Young’s modulus (surface biaxial modulus) within elasticity surface slice.

Besides surface elasticity, surface stress effect is another important factor which strongly influences the whole elastic properties of nanofilms. When top and bottom surface stresses are not equal each other i.e. the surface stresses are imbalance, the film will bend induced by the imbalance surface stresses. Surface stresses apply two effects on the film. One is force effect and the other is moment (torque) effect. The force expands (or compresses) the film and moment (torque) bends the film. The bending deformation and expansion (compression) deformation arise at the same time. They should influence each other and compete against each other. If surface stresses can be seen as isotropic, the bending curvature and expansion deformation (elongation strain) will be isotropic too. The stress and strain within the film are also isotropic at the same time. Therefore, the strain within the film can be described by

5 $$\varepsilon ={\varepsilon }_{0}-\frac{z}{R}={\varepsilon }_{0}-kz,$$

where *ε*_0_ is elongation strain (expansion deformation), *R* is radius of curvature and *k* = 1/*R* is bending curvature. The *z* direction is fixed along vertical direction while the reference plane (*x*-*O*-*y* plane) is fixed on the mid-plane of the film. The deformation of the bending film can be described by elongation strain *ε*_0_ and bending curvature *k* completely.

The elastic free energy of the film can be constructed by bulk elastic energy and surface elastic energy. There are two types of surface elastic energy of the films. One originates from the coupling between surface elasticity and surface strain (surface strain energy), and the other originates from the coupling between surface stress and surface strain (surface stress energy). The stress within surface slice can be obtained by

6 $$\sigma =\frac{1}{{t}_{s\sigma }}{\int }_{{t}_{s\sigma }}\sigma (z)dz=\frac{1}{{t}_{s\sigma }}\tau .$$

where surface stress is defined as

7 $$\tau ={\int }_{{t}_{s\sigma }}\sigma (z)dz.$$

Here, the stresses within stress surface slice are averaged for the sake of simplicity. Where *σ*(*z*) is the stress within stress surface slice, which is function of *z* coordinate. And *σ* is the average value of *σ*(*z*) within stress surface slice. The dimension of *τ* is N/m, and it is different from bulk material stress. While the dimension of *σ*(*z*) is Pascal (N/m^2^), which is same as bulk material stress.

Since the strain within the film is isotropic, the bulk elastic free energy can be expressed by

8 $$\frac{{E}_{el}}{V}=Y{\varepsilon }^{2}.$$

where *V* is the volume of the film, *Y* is biaxial modulus of the film, and it works the whole thickness *t* of the film. The top and bottom surface strain energies ${E}_{el}^{ts}$ and ${E}_{el}^{bs}$ are

9a $$\frac{{E}_{el}^{ts}}{{V}_{ts}}={Y}_{ts}{\varepsilon }_{ts}^{2},$$

9b $$\frac{{E}_{el}^{bs}}{{V}_{bs}}={Y}_{bs}{\varepsilon }_{bs}^{2},$$

respectively. Where *V*_ts_ and *V*_bs_ are volumes of top and bottom elasticity surface slices, *Y*_ts_ and *Y*_bs_ are surface biaxial moduli of top and bottom elasticity surface slices, respectively. Surface biaxial modulus modulates the actual modulus within elasticity surface slice and makes the modulus within elasticity surface slice be different from bulk like area. This surface elasticity effect influences the overall elastic property of nanofilms.

When the film bends or curls, the bulk elastic energy density can be obtained by the integral of whole film

10 $${E}_{el}=AY{\varepsilon }_{0}^{2}t+\frac{1}{12}AY{t}^{3}{k}^{2}.$$

The bulk elastic energy is dependent on elongation strain and bending curvature of the film. For a specific case that there is no bend of the film, the bulk elastic energy should be only dependent on the elongation strain.

Within the stress surface slice, surface stress energy density can be obtained by integration of stress and strain. Surface energy can be separated into two parts. One is induced by surface biaxial modulus, i.e. surface strain energy. Another is induced by surface stress, i.e. surface stress energy. The total surface energy can be obtained by integrating the surface elastic energy density and the surface stress energy density in their corresponding surface layers, respectively. Top and bottom surface energies *E*_ts_ and *E*_bs_ are

11a $${E}_{ts}=A{Y}_{ts}[{\varepsilon }_{0}^{2}{t}_{s}-{\varepsilon }_{0}k(t-{t}_{s}){t}_{s}+\frac{1}{3}{k}^{2}(\frac{3}{4}{t}^{2}-\frac{3}{2}t{t}_{s}+{t}_{s}^{2}){t}_{s}]+2A{\sigma }_{ts}({\varepsilon }_{0}{t}_{s\sigma }-\frac{1}{2}k(t-{t}_{s\sigma }){t}_{s\sigma }),$$

11b $${E}_{bs}=A{Y}_{bs}[{\varepsilon }_{0}^{2}{t}_{s}+{\varepsilon }_{0}k(t-{t}_{s}){t}_{s}+\frac{1}{3}{k}^{2}(\frac{3}{4}{t}^{2}-\frac{3}{2}t{t}_{s}+{t}_{s}^{2}){t}_{s}]+2A{\sigma }_{bs}({\varepsilon }_{0}{t}_{s\sigma }+\frac{1}{2}k(t-{t}_{s\sigma }){t}_{s\sigma }),$$

respectively. Where *σ*_ts_ and *σ*_bs_ are stresses within top and bottom stress surface slices. For the surface strain energy, the first, second and third terms are elongation strain, the coupling between elongation and bending strains, and the bending strain contributions, respectively. The elongation term is independent of film thickness due to the uniform property. In other words, the elongation strain is independent of *z* coordinate. The coupling and bending terms are dependent of film thickness due to the *z* position dependent property. The similar discussion suits surface stress energy. The total surface energy is the sum of the two surface energies, i.e. *E*_s_ = *E*_ts_ + *E*_bs_. While the total energy is the sum of bulk energy and surface energies, i.e. *E*_tot_ = *E*_el_ + *E*_s_.

Similar to bulk elastic energy, the surface energy (not only surface strain energy but also the surface stress energy) is also dependent on the elongation strain and bending curvature of the film. And if there is no bend of the film, the surface energy should be only dependent on the elongation strain.

The equilibrium state can be completely described by elongation strain and bending curvature. One can obtain these two parameters by using principle of minimum energy

12a $$k=\frac{6(t-{t}_{\sigma s})(Yt+{\Sigma }_{s}){\Delta }_{\tau }-6(t-{t}_{s}){\Delta }_{S}{\Sigma }_{\tau }}{[Y{t}^{3}+(3{t}^{2}-6t{t}_{s}+4{t}_{s}^{2}){\Sigma }_{S}](Yt+{\Sigma }_{S})-3{(t-{t}_{s})}^{2}{\Delta }_{S}^{2}},$$

12b $${\varepsilon }_{0}=\frac{3(t-{t}_{\sigma s})(t-{t}_{s}){\Delta }_{S}{\Delta }_{\tau }-[Y{t}^{3}+(3{t}^{2}-6t{t}_{s}+4{t}_{s}^{2}){\Sigma }_{s}]{\Sigma }_{\tau }}{[Y{t}^{3}+(3{t}^{2}-6t{t}_{s}+4{t}_{s}^{2}){\Sigma }_{s}](Yt+{\Sigma }_{s})-3{(t-{t}_{s})}^{2}{\Delta }_{S}^{2}},$$

where

13 $$\begin{array}{cc}{\Delta }_{{\rm{\tau }}}={\tau }_{ts}-{\tau }_{bs}, & {\Sigma }_{{\rm{\tau }}}={\tau }_{ts}+{\tau }_{bs},\\ {\Delta }_{S}={S}_{ts}-{S}_{bs}, & {\Sigma }_{S}={S}_{ts}+{S}_{bs},\\ {S}_{ts}={t}_{ts}{Y}_{ts}, & {S}_{bs}={t}_{bs}{Y}_{bs}.\end{array}$$

For the sake of simplicity, it can be assumed that the thickness of stress surface slice is same as that of elasticity surface slice, i.e. *t*_*σ*s_ = *t*_s_. And then Eq. (12) can be decayed as

14a $$k=\frac{6(t-{t}_{s})[(Yt+{\Sigma ^{\prime} }_{s}){\Delta }_{\tau }-6{\Delta }_{S}{\Sigma }_{\tau }]}{[Y{t}^{3}+(3{t}^{2}-6t{t}_{s}+4{t}_{s}^{2}){\Sigma }_{S}](Yt+{\Sigma }_{S})-3{(t-{t}_{s})}^{2}{\Delta }_{S}^{2}},$$

14b $${\varepsilon }_{0}=\frac{3{(t-{t}_{s})}^{2}{\Delta }_{S}{\Delta }_{\tau }-[Y{t}^{3}+(3{t}^{2}-6t{t}_{s}+4{t}_{s}^{2}){\Sigma }_{s}]{\Sigma }_{\tau }}{[Y{t}^{3}+(3{t}^{2}-6t{t}_{s}+4{t}_{s}^{2}){\varSigma }_{s}](Yt+{\Sigma }_{s})-3{(t-{t}_{s})}^{2}{\Delta }_{S}^{2}}.$$

For the case of the relatively thinner surface slice (or the relatively thicker film), surface slice thickness is much less than whole film thickness. The surface slice thickness can be neglected, i.e. *t*_s → _0, but surface effects cannot be neglected, the equilibrium state can be described by

15a $$k=\frac{6t{\varDelta }_{\tau }(Yt+{\Sigma }_{S})-6t{\Sigma }_{\tau }{\Delta }_{S}}{(Y{t}^{3}+3{t}^{2}{\Sigma }_{S})(Yt+{\Sigma }_{S})-3{t}^{2}{\Delta }_{S}^{2}},$$

15b $${\varepsilon }_{0}=\frac{3{t}^{2}{\Delta }_{S}{\Delta }_{\tau }-[Y{t}^{3}+3{t}^{2}{\Sigma }_{s}]{\Sigma }_{\tau }}{[Y{t}^{3}+3{t}^{2}{\Sigma }_{s}](Yt+{\Sigma }_{s})-3{t}^{2}{\Delta }_{S}^{2}}.$$

Equations (15) represent the consistent results with Sadeghian *et al*.^35^. But Sadeghian *et al*. didn’t give the algebraic solution. If the condition is simplified further, when top and bottom surface elastic parameters are equivalent, i.e. *S*_ts_ = *S*_bs_ = *S*, *∆*_*S*_ = 0, and *∑*_*S*_ = 2 *S*, Eq. (15) can be simplified as

16a $$k=\frac{6{\Delta }_{\tau }}{Y{t}^{2}+6tS},$$

16b $${\varepsilon }_{0}=-\,\frac{{\Sigma }_{\tau }}{(Yt+{\Sigma }_{S})}.$$

Liu *et al*. modified the Stoney formula^17^ and gave the same result as Eq. (16a).

## Results and Discussions

With the decreasing of film thickness, the relative number of atoms that are bonded surfaces increases. And then, surface stress and surface elasticity effects strongly affect the overall mechanical properties especially elastic property of nanofilms and cannot be neglected any more^36^. Van der Waals bond outside the film absents and broken bond arises. Dangling bonds combine together and the combination induces surface atoms to move. The movement allows surface atoms to depart from their customary position and apply stress on film surface. This surface stress effect may exist at inner area near surface and this area can be called as stress surface slice. At the same time, inter-atomic forces and lattice structure near surface are changed. This surface procedure changes the Young’s modulus (as well as biaxial modulus) near surface. Similar to surface stress, the surface elasticity may also exist at inner area near surface and this area can be called as elasticity surface slice. The surface stress induces initial strain of the equilibrium of nanofilms and surface elasticity influences this effect. The initial strain along vertical direction is different from the in-plane directions due to Poisson’s effect of the film. For example, surface stress compresses the film along in-plane directions while the vertical direction expands via Poisson’s effect. This equilibrium property makes lowered symmetry of nanofilms^33^. If top and bottom surface stresses are not balance, the nanofilm will bend even curl into a nanotube^27^. The biaxial modulus *Y* = (*c*_11_ + 2*c*_12_)(*c*_11_ × *c*_12_)/*c*_11_ and Poisson’s ratio *v* = *c*_12_/(*c*_11_ + *c*_12_)^36^. The vertical direction strain can be obtained by *ε*_z_ = −2*vε*/(1-*v*). The ratio between vertical and in-plane direction lattice constants *Δ* = (1 + *ε*_z_)/(1 + *ε*). The bulk and surface parameters of nanofilms in this paper are shown in Table 1.

**Table 1:** Bulk elastic constants^21^, surface elasticity, surface stress and surface slice thickness of nanofilms.

	*c*_11_(*c*_22_) (GPa)	*c*_12_ (GPa)	*τ*_ts_ (*τ*_bs_) (N/m)	*S*_ts_ (*S*_bs_) (N/m)	*t*_s_ (nm)
Cu	70.04	32	0.95	2.5	0.25
Ni	142.56	52.16	1	0.2	0.25
Ag	64.8	26.88	0.6	10	0.25
Au	45.44	21.12	1.1	8.5	0.25
Pd	62.24	28.48	1.58	33	0.25
Pt	56.8	27.04	1.7	13	0.25

Intrinsic surface stress induces initial strain along in-plane direction, in other words, intrinsic surface stress changes the inter-atomic distance (lattice constant). This procedure is just the relaxation of overall film. Since elastic constant of material is very sensitive to inter-atomic distance *d*^30,31^, the overall elastic property of nanofilms should be changed by this surface effect. On the other hand, the lattice constant along vertical direction changes via Poisson’s effect. In consideration of the rule of Poisson’s effect, the vertical relaxation procedure should be inversely. For example, if the in-plane direction is expanded (compressed) by intrinsic surface stress, the vertical direction will be relaxed inwards (outwards)^33^.

**Figure 2:**
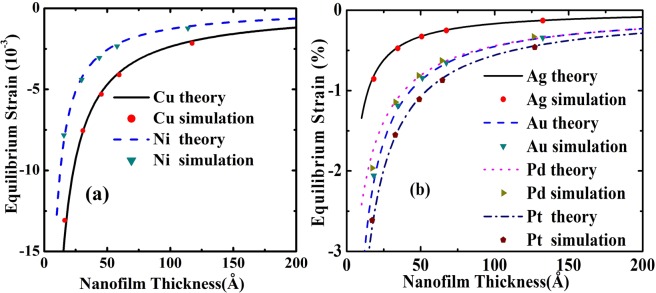
Equilibrium strains. The result lines were calculated by Eq. (14b). The details of MD calculation see ref.^21^. (**a**) Equilibrium strains of Cu and Ni nanofilms. (**b**) Equilibrium strains of Ag, Au, Pd and Pt nanofilms.

In Fig. 2, one can easily find that equilibrium strain of nanofilms is obviously dependent on film thickness. The lines are present theoretical calculations. The applications of our theoretical scheme to Cu, Ag, Au, Ni, Pd, Pt nanofilms showed good agreement with MD calculations. There are two aspects about the change regulation of equilibrium strain lines of these nanofilm materials. The first factor is that it is more difficult to compress (expand) a thicker film. Since the intrinsic surface stress is independent from film thickness, the equilibrium strain should be smaller for a thicker film. This thickness dependence is obvious and is primary factor. Another factor to influence the equilibrium strain is surface elasticity effect. The surface elastic constant is independent from film thickness, but it’s influence on the overall elasticity is dependent on the film thickness. Surface elasticity effect influences the equilibrium strain of the film obviously when film thickness is only several nanometers. The lager biaxial modulus as well as the smaller intrinsic surface stress induces the smaller equilibrium strains for the same film thickness. The smaller biaxial modulus and the larger intrinsic surface stress of Pt induce largest equilibrium strain comparing with other nanofilms for the same film thickness. The compress surface stress makes atomic be close to each other and the lattice constant to be smaller. The smaller the inter-atomic distance, the stronger the inter-atomic force. Stronger inter-atomic force certainly means lager elastic constant. And then, Young’s modulus (biaxial modulus) is enhanced by surface effects. As additional Young’s modulus (biaxial modulus), the numerical value of surface elasticity should be positive.

**Figure 3:**
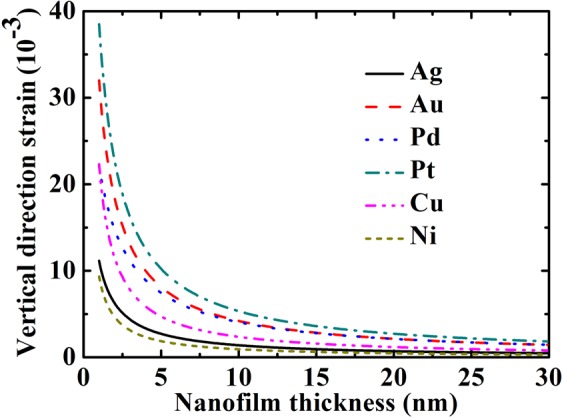
The vertical direction strains of Ag, Au, Pd, Pt, Cu and Ni nanofilms. The bulk and surface parameters were all shown in Table 1.

Figure 3 showed the vertical direction strains of Ag, Au, Pd, Pt, Cu and Ni nanofilms. The in-plane direction strain is induced by intrinsic surface stress. Vertical equilibrium strain is induced by Poisson’s effect and has different sign comparing to in-plane direction strain.

**Figure 4:**
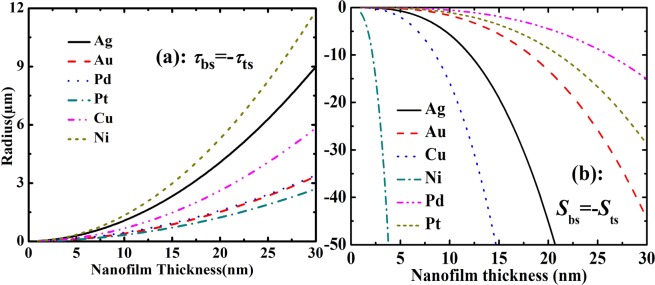
The radius *R* of Ag, Au, Pd, Pt, Cu, and Ni curled nanofilms. (**a**) Surface elasticity and top surface stress were shown in Table 1, and bottom surface stress was set as *τ*_bs_ = −*τ*_ts_. (**b**) Surface stress and top surface elasticity were shown in Table 1, and bottom surface elasticity was set as *S*_bs_ = −*S*_ts_.

**Figure 5:**
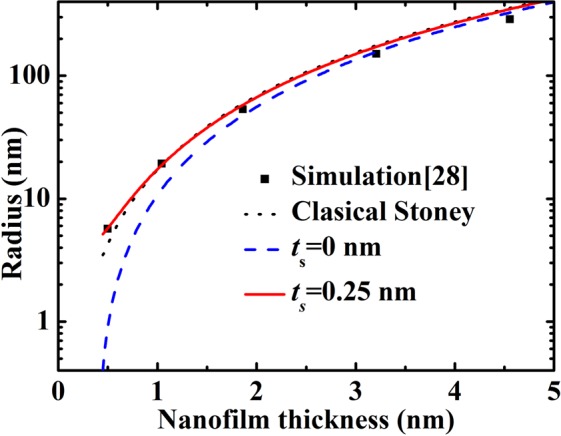
The radius *R* of Si nanotube. The details of MD calculation for bending radio of Si nanotube see ref.^27^.

Figures 4(a) and 5 showed that when surface stress is imbalance, the nanofilm will bend even curl into a nanotube. The bottom surface stress is set as minus i.e. *τ*_bs_ = −*τ*_ts_, for example (while keep top surface stress remaining unchanged and as shown in Table 1). In Fig. 4(a), the changes law of different nanofilm materials is same as Fig. 2. The larger in-plane strain induces a larger vertical strain, and vice versa. In Fig. 5, the top and bottom surface stresses are 0.40 and −1.34 N/m, top and bottom surface elasticities are −18.35 and −0.339 N/m. The classical Stoney formula is usually applied to calculate a plate bending induced by surface stress (The surface stress in classical Stoney formula is induced by coating, it is different from intrinsic surface stress), but it does not contain surface elasticity^37^. Hence, the absence of surface elasticity means that classical Stoney formula should be modified by surface effects (surface elasticity effect especially). If the elasticity surface slice thickness is neglected, the theoretical calculation will not give agreement with MD simulation when Si nanofilm thickness is smaller than 2 nm. If the elasticity surface slice thickness is set as *t*_s_ = 0.25 nm, the theoretical line showed a good agreement with MD simulation (see in Fig. 5). This fact shows that the surface slice thickness influences elastic properties of ultrathin nanofilms which are only several nanometers strongly. And the importance of surface slice thickness is enhanced by smaller film thickness. On the other hand, even between top and bottom surface stresses is balance (i.e. Δ_*τ*_ = 0), the bend may also appear. And then, the nanofilm is bent by surface elasticity imbalance, as shown in Fig. 4(b). The small surface elasticity and large bulk elasticity of Ni induce a small bending curvature i.e. large bending radius.

**Figure 6:**
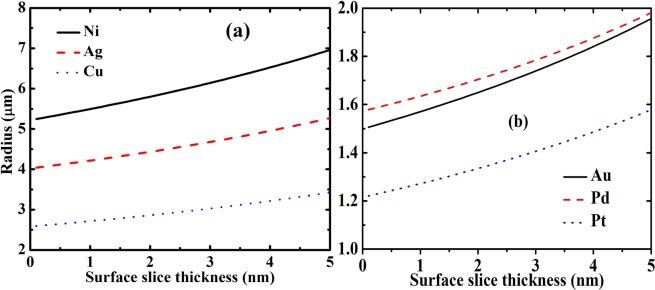
The influence of surface slice thickness on the radius of curled nanofilm. The bottom surface stress was set as *τ*_bs_ = −*τ*_ts_. The other surface parameters were set to be constant as shown in Table 1. (**a**) Radius of curled Ni, Ag and Cu nanofilms. (**b**) Radius of curled Au, Pd and Pt nanofilms.

The positive surface elasticity leads to nanofilms be difficult to bend and largen the bending radii of curled nanofilms. The existence of elasticity surface slice thickness means that part of surface elasticity close to mid-plan and away from surface. The surface elasticity has a greater impact on bending curvature when it is farther from mid-plane and vice versa. Therefore, the existence of elasticity surface slice thickness weakens surface elasticity effect on the bending film. And then, the elasticity surface slice thickness makes the radius be smaller. On the other hand, the larger stress surface slice thickness induces part of surface stress to be close to mid-plane. And then the surface stress applies smaller moment on the nanofilm. Therefore, the larger stress surface slice thickness gives smaller bending curvature i.e. larger radius. In Eq. (14a), the hypothesis of *t*_*σ*s_ = *t*_s_ reveals the competition between elasticity and stress surface slice thickness effects. Since the surface stress is the primary factor on the bending film, the bending radius tends to largen with the surface slice thickness increasing, as shown in Fig. 6.

**Figure 7:**
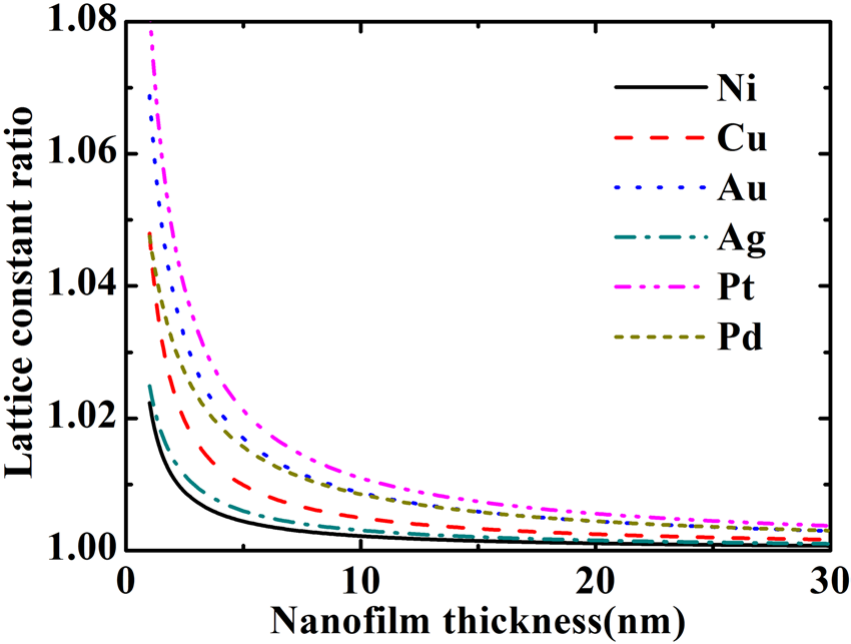
The lattice constant ratio between vertical and in-plan directions.

Surface stress compresses (expands) the nanofilm along in-plan direction, while vertical direction should be expanded (shrunk) via Poisson’s effect. Therefore, there is lattice constant difference between vertical and in-plane directions. The ratio of lattice constants between these two directions was shown in Fig. 7. The compressed in-plane strains of these nanofilm materials induce expansion strains along vertical direction. Therefore, the ratio of vertical and in-plane direction lattice constants is larger than 1. The lattice constant difference between in-plane and vertical directions introduces lowered symmetry of nanofilms. This lowered symmetry introduces an additional elastic constant which is no bulk counterpart^28,33^. And this additional elastic constant relates to the interactions of expansion and vertical lattice relaxation. This coupling characteristic is similar with the volume dependence *c*/*a* in the hexagonal crystal materials^28^. Since the vertical direction always behaves inverse relaxation comparing to in-plane directions, the additional surface elastic constant has different sign from other surface elastic constants^33^. This symmetry lowering effect is derived mathematically, but not only qualitative analysis here. Figure 7 showed that lattice constant ratio is dependent on film thickness. The larger film thickness results in smaller lattice constant ratio, which means weakened symmetry lowering effect. The relatively larger biaxial modulus of Ni indicates that Ni nanofilm is harder and is difficult to be compressed. This induces small in-plane equilibrium strain as well as small vertical equilibrium strain, the lattice constant ratio of Ni to be relatively smaller. Surface stress of Ag is obviously smaller than other films. Therefore, the lattice constant ratio of Ag is also relatively smaller despite the smaller biaxial modulus. On the other hand, due to the large surface stress, Pt nanofilm has the largest lattice constant ratio in these six materials. Au has a relatively larger lattice constant ratio due to it’s small biaxial modulus.

## Conclusions

This work researched self equilibrium strain, bending (curling) and symmetry lowering of nanofilms. Established the corresponding theoretical scheme which contains surface elasticity and surface slice thickness effects. The present theory was used to compare with Cu, Ni, Au, Ag, Pt and Pd nanofilm self equilibrium strain simulations, and was used to compare with the simulation of Si nanofilm curling problem. When the film elongated by balance intrinsic surface stress (the surface elasticity is also balance), there is only identical elongation strain but no bending strain. The strain within film is identical anywhere. The surface slice thickness is no influence on this condition. When nanofilm is bent, the strain within nanofilm varies with vertical direction (*z* coordinate). The same surface elastic constant with different *z* coordinate gave different additional elastic energies when nanofilm bent. On the other hand, the same surface elastic constant with different *z* coordinate gave different contributions to the bending curvature. And then the surface slice thickness influences the bending curvature of nanofilms. The symmetry lowering of nanofilms is derived mathematically, but not only qualitative analysis in this paper. Theoretical arithmetic showed that symmetry lowering effect is dependent on film thickness obviously.
